# Foreign Bodies in the Facial Orifices

**Published:** 1907-03

**Authors:** Richard R. Daly

**Affiliations:** Atlanta, Ga.


### FOREIGN BODIES IN THE FACIAL ORIFICES.*

By Richard R. Daly, M. D.

To brush a speck from the eye or to pick a bone or pin from the throat is a common experience, unless one thinks of the still more common one of finding that the speck is gone or the bone swallowed by the time the patient gets to him. In either event the foreign body case is not welcome, as a rule. So large a percentage of them come at night that one wonders if an obstetrical practice could be much worse.

The moral aspect of the question is of at least momentary interest. The speck that has been in the eye all day or the pin that has been in the throat since supper-time, becomes, along about midnight, a sort of anti-conscience. It won't let the patient sleep till morning, and it drives him to the doctor always without a cent of money in his pocket. A speck or bone once out means oblivion not so much to pain as to paying. A dozen night calls will usually net twelve unpaid bills.

Those who intend to pay go to the doctor directly they are injured, and that is in the day-time. To these patients we frequently give instant and immense relief, but sometimes the problem is of grave importance.

I used to think that every school child should be taught to evert the lids as soon as he was old enough to study physiology, and have this equipment in his first aid to the injured course; but a little

*Read before the Fulton County Medical Society, Feb. 12th, 1906.

knowledge is a dangerous thing around the eye as elsewhere, and now it is a question as to whether trained nurses are competent to do this simple thing and stop there.

So many specks are found beneath the upper lids that we look there first for eye difficulties. They are easily removed with a corner of a handkerchief or bit of rolled cotton. Sometimes they are sharp and cause the patient so much pain and lachrymation that he rubs and floods them out without knowing it. The sharp angles may scratch the conjunctiva or cornea and the sensation of the foreign body remains.

At the time of the examination the small scratch can usually be seen. It is a help to tell the patient not to indicate in what part of the eye the body seems to be till one has either removed it or seen the wound it has made. Touching the latter lightly will cause the patient to say, "That's it; take it out." One knows then that he has probably found the seat of the trouble. There are still the fossae to examine to make sure, and this should always be done. It requires practiced technic and should not be attempted otherwise.

When bodies are embedded in the cornea, we have a surgical affair of more importance because of the danger of subsequent ulceration, depending upon the depth of the injury and the septic character of the object. These bodies are frequently hard to see. Cross illumination should never be omitted, and the depth of the injury should be known before the body is touched.

It is a common thing among grinders and metal workers for bits of hot steel or iron to become embedded in the cornea, and when this is removed to have a brown, rust-colored spot remaining. This colored spot is often dug at by fellow workmen. Some of the men acquire great skill in removing things, but, unfortunately, they can't let well enough alone. The rust spot will come away in a day or two, or may be brushed out after that time without trouble.

The immense magnets with which some oculists are equipped are interesting and, I think, useless and dangerous devices for getting out metal specks. They never draw out a speck that can not be as readily removed by an instrument under finger control, and they have been known to make the speck jump so quickly as to cause unnecessary injury.

The tolerance of the eye is remarkable when one thinks of the delicacy of its structure. How bravely it stands up under all the old women's devices of flax-seeds, eye-stones, tea leaves, etc. It is a wonder that some generations have any eyes left. A clinical case will illustrate this.

A train robber and would-be murderer received a forty-nine-year sentence for his sins when he was twenty-seven years old. It meant life imprisonment to him, and he became rebellious and showed clearly the insanity which doubtless was the basis of his bizarre crimes. He was sent to the insane wards of the prison, where he apparently got better and from which he was returned to his cell. There and while at work he secured some needles, a piece of shingle, some string and a weight. He put the needles through the shingle, letting them project half an inch; he weighted the board, suspended it by the string, calculated where it would drop if released and lying down, he placed his eyes in that position. He released the string and received the needles in the eyes through the tarsal cartilages. His cries brought assistance, and he was returned to the insane ward for treatment. He could not help looking downward (toward his feet) when the impact was coming, so no great harm was done. The slight punctures healed quickly the cornea was not involved.

Over a year later and during which time his life had been troublesome and he had made several attempts at escape, he made another attack upon his eyes. In the lavatory early one evening he struck and broke an electric bulb. The noise called the attendants immediately and he was searched for broken glass. Several small bits were taken from him and his mouth was examined, but nothing was found there. He was shut in his room for the night, as usual. The next morning the attendants were frightened to see what they thought was part of his eyes running out on his cheeks. It transpired that he had secreted a piece of glass in his mouth that lay so close to his cheek as to evade the attendant's finger. This glass he had broken into small bits, wrapped them in a little piece of toilet paper, run a thread through the paper so as to cut it when pulled, and then put such a package under each eyelid. After the paper softened with the tears, he held it with one hand and pulled the thread loose with the other. The paper pulp seen in the morning led the attendants to think it was vitreous. Many small pieces of glass had been liberated and were lodged in the conjunctival folds. The conjunctiva was torn in several places. Eighty odd pieces of glass were removed, the shreds of conjunctiva replaced, the eye bandaged for a day, and complete recovery followed in a few days more. Then the patient began rubbing food, butter and salt into his eyes. He gave this explanation: He was looked upon as a dangerous man, and a certain transportation company would never permit him to be pardoned. The length of his sentence meant life imprisonment. If, however, he were blind, he could not be dangerous as a train robber and he might get a pardon. He would rather be blind and free than have his vision and be a prisoner. His syllogism had but two terms and would not work. He continued in assaulting his eyes until he became totally blind, and he is now an insane prisoner. But before the blindness came, the eyes had withstood an incredible amount of injury without even corneal ulceration.

Foreign bodies in the eye occasionally assume curious positions. A man complained of having been struck in the eye by something when chopping a board. The small traumatism showed half-way to the outer canthus, but nothing causing it was found. The vitreous was full of blood, and it became advisable to ennucleate the eye. The operation proceeded to the point of cutting the nerve, when it was found the scissors would not close. A dissection discovered a piece of nail driven into the nerve and through the foramen. At its external entry there was no escape for vitreous, and we had no idea of its presence till the scissors struck it.

Sometimes the offending substance is hot or caustic, and a serious surgical condition follows. It is of importance to tell workmen and others liable to such injuries that the best first aid is sweet oil, freely used. I would emphasize the value of the dissemination of this information, because it so often has prevented serious injury from the use of other things, such as mud and saliva, tobacco juice, bird's entrails, or any other disgusting thing that can be thought of. In the making of many good old home remedies, the imagination ran riotous with filth as a companion, and the results were horrible.

Perhaps it was with this sort of training that an insane patient of mine manifested a penchant for toads. She always carried one when she could and liked to hand them to people or do bizarre things with them herself. She put a little one in her mouth one day and we thought she had swallowed it till her pulling at her mouth showed she was in difficulty. In some way the small toad had lodged back of the soft palate and, being alive, engaged itself in the nasopharynx, where the patient's efforts could not dislodge it. Neither could mine till chloroform quieted her, and forceps delivery in axis traction followed. The toad was alive.

Patients of the demented class were frequently filling their mouths so full of food as to choke and need assistance. Then it was to take one's chances of being bitten and infected, because the index finger in a hurry is the only thing that will clean them out.

Most cases that come to our offices for foreign body in the throat have had it there and have dislodged it to pass into the stomach. But the resulting scratch hurts just as much and misleads them. It requires careful examination and then much tact on the doctor's part to reassure such a patient and keep him in faith through the uncomfortable night that follows. I know how I insisted on an X-ray examination of my own throat concerning a small piece of chicken bone that had lodged there and passed on.

One should always inspect the laryx first in such cases and then palpate everything with the finger. It will go farther and tell more of the facts and the usual places of enlodgment than any other instrument, and it is the best thing to remove objects with. A little pressure will make the finger nail somewhat prehensile, and the object can be dragged loose and out, if found.

In the larynx I once saw a case of too much confidence. A young school girl complained to a doctor that she had a small stick-pin in her throat. She was holding it in her mouth and laughed, the inspiration drew it in, she thought. He pretended to use a mirror, and told her she must have coughed it out again, for it wasn't to be found. She went to school all the next day, and in the second I saw her because her voice was husky and pain increasing. A mirror instantly showed the pin. It was sticking through the epiglottis from below upward. The head was out of sight in the trachea. I reached the shank with a McKenzie forceps, thrust it further down the trachea to release the point from the epiglottis, and then extracted it from the larynx. At that instant the patient, who had been quiet, struck my hand and made me drop the pin in the pharynx. She swallowed it and we found it in her stool the third day. It was one and three-fourth inches long. Another instance of strange tolerance.

As if to offset this case, another came, giving the history that she was in the habit of holding many pins in her mouth and thought that she had breathed one into her larynx. She was easily examined and no pin or redness was discovered. She could not be reassured, and in a few days of great agitation, during which she was continually grasping and pressing the larynx, she was in such a deplorable state mentally and the parts were so injured by her handling, that it was counselled to operate. The larynx and cricoid were laid completely open and nothing was discovered. The wound was closed and when she recovered consciousness a pin was shown her as coming from the part. She recovered uneventfully.

There are classic cases of teeth, coins, and many other things in the larynx, but they are none of them in my experience.

Children put many strange things in their noses, such as buttons, stones, toys, hairpins—anything they can think of at the time; and their extraction is sometimes a matter of considerable skill. It is usually best to get them out the way they went in, but one should not forget there are posterior nares, and not one chance in a thousand of the small object causing trouble if it is pushed out that way.

In the nose, too, there are tolerance and intolerance amusingly shown. A traveling man came to me for some nasal trouble, and I discovered a small gauze packing that had been put there over a month before. He hadn't returned to the surgeon who placed it there and he forgot it.

On the other hand, it is my custom to pack the nose with long, slender pledgets of cotton, saturated with an anæsthetic or supra-renal extract in preparation for an operation. These were removed and an operation performed in a certain case. Next morning the lady said she had not slept at all because one of those pledgets had been left there. Examination showed a clear field, but having had experience with my laryngotomy case, I managed to find a pledget somewhere, take it from her nose and offer sincere apologies for the unwarranted intrusion upon her nasal privacy. Since then I have always counted the inserted pledgets out loud when putting them in and called the patient's attention to the same number when I removed them. It saves legedermain later, not to speak of a clear conscience.

In the ear we find various articles, put there by children or insane people; and sometimes they are forced through the isthmus of the canal and are difficult of extraction. One should always be careful in removing impacted cerumen, because it may have some foreign body as a nucleus. I remember finding several horse-flies surrounded by wax in a boy's ear. They seemed never to bother him till the occlusion caused deafness. Yet what is more troublesome to most patients than to have a live insect get into the ear? In their distress they resort to almost any means of relief suggested, and sometimes injure themselves. I know of nothing better for first aid than grain alcohol or whiskey. Either will kill the insect, disinfect the parts and while it may sting if there are abrasions, it won't do any harm and the patient comes to the doctor's hands in good condition for further treatment.

An interesting case of tolerance in the ear came to my attention one day in February. Large masses of impacted cerumen occluded the canal and made the young man deaf. Gentle douching with mild alkaline solution removed some of it and disclosed a grayish fibrous mass beyond the isthmus. A small forceps removed a shred, which proved to be cotton, and then stronger traction removed a large wad of this material. A similar thing was found in the other ear. The patient explained that he had put cotton in his ears when swimming during the previous September and must have forgotten it. No injury had been done to the parts.

Though several instances of great tolerance have been shown, it would be a mistake to say that foreign bodies could be handled carelessly. Unless great care and precision are exercised in their removal, there are likely to be very unpleasant results, no matter how insignificant they may appear in the beginning.

1008 English-American Building.

